# Identification and validation of disulfidptosis-associated molecular clusters in non-alcoholic fatty liver disease

**DOI:** 10.3389/fgene.2023.1251999

**Published:** 2023-09-08

**Authors:** Xiaoxiao Yu, Zihao Guo, Zhihao Fang, Kai Yang, Changxu Liu, Zhichao Dong, Chang Liu

**Affiliations:** Department of General Surgery, Fourth Affiliated Hospital of Harbin Medical University, Harbin, China

**Keywords:** non-alcoholic fatty liver disease, disulfidptosis, molecular clusters, immune infiltration, machine learning, prediction model

## Abstract

**Objective:** Non-alcoholic fatty liver disease (NAFLD) is the most prevalent liver disease in the world, and its pathogenesis is not fully understood. Disulfidptosis is the most recently reported form of cell death and may be associated with NAFLD progression. Our study aimed to explore the molecular clusters associated with disulfidptosis in NAFLD and to construct a predictive model.

**Methods:** First, we analyzed the expression profile of the disulfidptosis regulators and immune characteristics in NAFLD. Using 104 NAFLD samples, we investigated molecular clusters based on differentially expressed disulfidptosis-related genes, along with the related immune cell infiltration. Cluster-specific differentially expressed genes were then identified by using the WGCNA method. We also evaluated the performance of four machine learning models before choosing the optimal machine model for diagnosis. Nomogram, calibration curves, decision curve analysis, and external datasets were used to confirm the prediction effectiveness. Finally, the expression levels of the biomarkers were assessed in a mouse model of a high-fat diet.

**Results:** Two differentially expressed DRGs were identified between healthy and NAFLD patients. We revealed the expression profile of DRGs in NAFLD and the correlation with 22 immune cells. In NAFLD, two clusters of molecules connected to disulfidptosis were defined. Significant immunological heterogeneity was shown by immune infiltration analysis among the various clusters. A significant amount of immunological infiltration was seen in Cluster 1. Functional analysis revealed that Cluster 1 differentially expressed genes were strongly linked to energy metabolism and immune control. The highest discriminatory performance was demonstrated by the SVM model, which had a higher area under the curve, relatively small residual and root mean square errors. Nomograms, calibration curves, and decision curve analyses were used to show how accurate the prediction of NAFLD was. Further analysis revealed that the expression of three model-related genes was significantly associated with the level of multiple immune cells. In animal experiments, the expression trends of DDO, FRK and TMEM19 were consistent with the results of bioinformatics analysis.

**Conclusion:** This study systematically elucidated the complex relationship between disulfidptosis and NAFLD and developed a promising predictive model to assess the risk of disease in patients with disulfidptosis subtypes and NAFLD.

## Introduction

Non-alcoholic fatty liver disease (NAFLD), the most prevalent type of liver disease in the world, is a metabolic syndrome that may progress from simple liver steatosis to non-alcoholic steatohepatitis, which increases the risk of developing cirrhosis and cancer (Powell et al., 2021). The prevalence of NAFLD is approximately 29.62% over all of Asia, according to studies (Li et al., 2019). It is also now recognized as a multisystem metabolic illness, and it is closely linked to a higher risk of cardiovascular disease and chronic kidney disease (Cai et al., 2020; Li et al., 2022). More importantly, the most widely accepted “multiple strikes” theory does not yet fully explain the disease mechanism; as a result, the most effective approaches to treating and preventing NAFLD currently focus more on reducing the patient’s body mass and improving diet and lifestyle (Lin and Kohli, 2020; Pugliese et al., 2022). An increasing number of biomarkers have been linked to NAFLD recently, but the results may not be conclusive because of limited sample sizes or individual data sets (Jiang et al., 2021; Zeng et al., 2021). Therefore, it would be crucial for therapeutic purposes to further precisely identify the molecular subtypes of NAFLD at the molecular level and to create multivariate prediction models.

A recent investigation by Liu et al. discovered a new form of cell death: disulfidptosis (Liu et al., 2023). Disulfide bonds play a significant role in the creation and breakdown of actin cytoskeleton proteins, and changes in the redox status of cells are linked to the occurrence of disulfidptosis, which can induce cell death by changing the configuration of cytoskeletal proteins (Zheng et al., 2023). Importantly, oxidative stress accelerates the development of NAFLD by activating several transcription factors (Tu et al., 2019), promoting the activation of hepatic stellate cells, macrophages, and Kupffer cells, exacerbating inflammation, fibrosis, and apoptosis in NAFLD (Pan et al., 2020). Additionally, insulin resistance and NAFLD disease development are also highly correlated with sulfide metabolism (Dhamija et al., 2009; Lu et al., 2022). As a result, we postulated that disulfidptosis is likely linked to the emergence of NAFLD. Therefore, it may be possible to explain NAFLD by better understanding the molecular properties of the disulfidptosis-related genes (DRGs).

In our study, we extensively analyzed the immune microenvironment and the differentially expressed disulfidptosis-related genes (DE-DRGs) in NAFLD patients and controls. We further separated NAFLD patients into two clusters associated with disulfidptosis based on DE-DRGs and evaluated the immune cell disparities between the two clusters. The biological processes and pathways of enrichment were then elucidated using cluster-specific differentially expressed genes (DEGs), which were discovered using the Weighted gene co-expression network analysis (WGCNA) algorithm. Additionally, by evaluating the discriminative performance of four machine learning algorithms, a predictive model was created that revealed patients with different molecular clusters. We used the model genes to construct the nomogram while calibration curve and decision curve analysis (DCA) were used to demonstrate the predictive power of the nomogram. Receiver operating characteristic (ROC) curve analysis was performed on two external datasets to validate the diagnostic value of the diagnostic models. More importantly, three genes were highly correlated with the formation of disulfidptosis-associated clusters, and the results of GSEA analysis showed that they may be involved in NAFLD formation by inhibiting the anti-oxidative stress pathway. Disulfidptosis occurs when cells undergo disulfide stress due to glucose deficiency or oxidative stress (Hu et al., 2023). DDO, FRK, and TMEM19 are likely to be involved in disulfidptosis via this pathway thereby advancing NAFLD progression. To strengthen the case for our study, we further validated the expression of three model-related genes in the high-fat diet (HFD) mouse model.

## Material and methods

### Data collection

**FIGURE 1:**
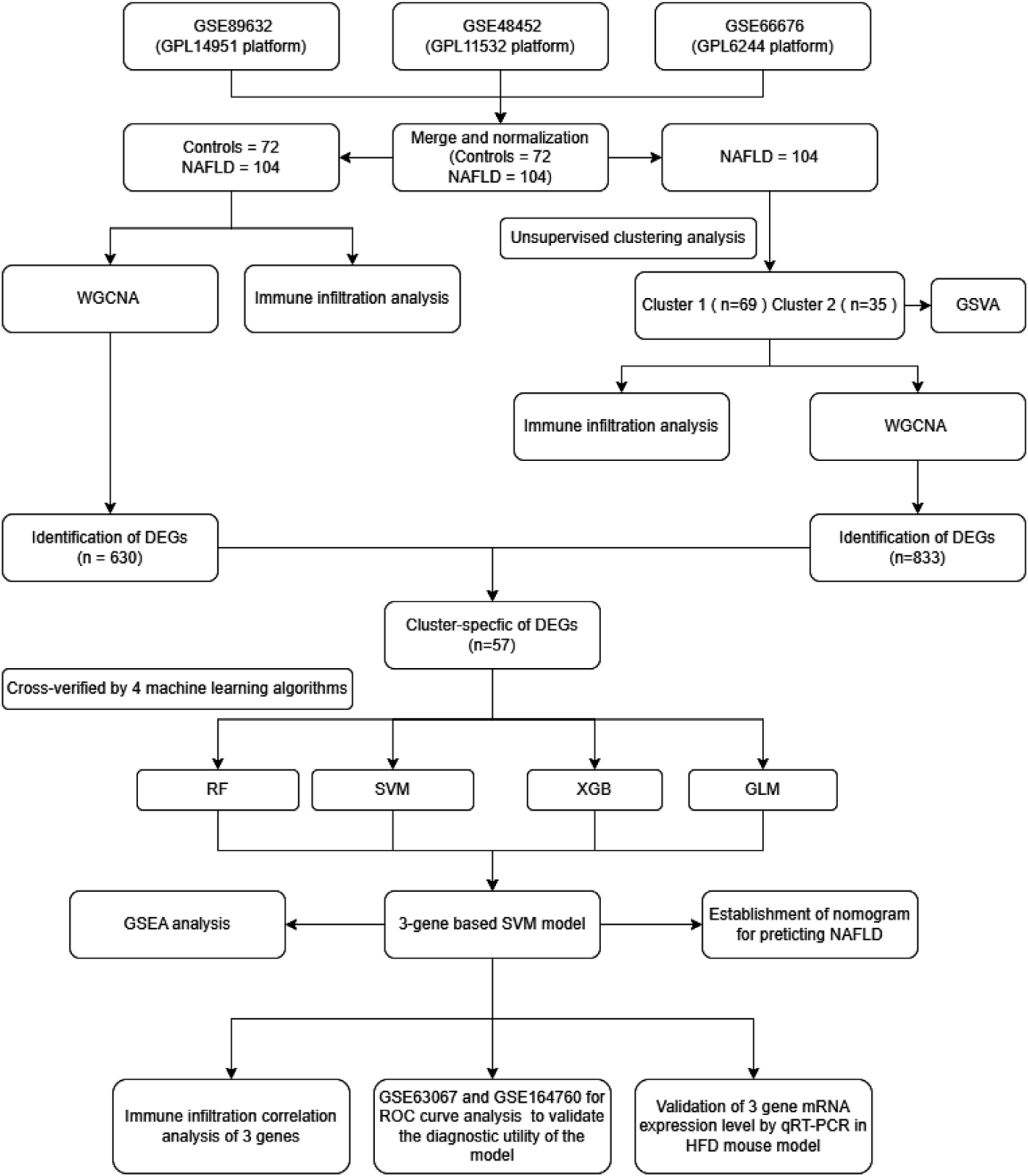
Research flow chart.

The study’s flow chart is shown in Figure 1. We conducted a systematic search of the Gene Expression Omnibus database (GEO) (http://www.ncbi.nlm.nih.gov/geo/) using the terms: “*Homo sapiens*” and “NAFLD”. This study included datasets that met the following eligibility criteria: 1) the type of study was expression profiling by array; 2) the dataset contained liver tissue samples from NAFLD patients and controls; 3) the sample size was greater than 15; 4) the raw data or array gene expression profiles were available in the GEO. Finally, five datasets were identified from the GEO database. GSE89632 (Microarray, platform GPL14951) (Arendt et al., 2015), GSE48452 (Microarray, platform GPL11532) (Ahrens et al., 2013), GSE66676 (Microarray, platform GPL6244) (Xanthakos et al., 2015), GSE63067 (Microarray, platform GPL570) (Frades et al., 2015) and GSE164760 (Microarray, platform GPL13667) (Pinyol et al., 2021)were included in this study.

### Datasets analyses

First, we combined the datasets GSE89632, GSE48452, and GSE66676, which had 72 normal samples and 104 NAFLD samples. Then, the merged gene expression datasets were normalized by using the “sva” package (Leek et al., 2012). DEGs between the NAFLD and control groups were discovered using the “limma” package (Colaprico et al., 2016). *p*-values lower than 0.05 were regarded as statistically significant. The datasets GSE63067, including 14 normal samples and 32 NAFLD samples, and GSE164760 including 6 normal samples and 74 NAFLD samples, were used to be the validation set. In the Liu et al. research, ten genes were connected to disulfidptosis (Liu et al., 2023). GYS1, NDUFS1, OXSM, LRPPRC, NDUFA11, NUBPL, NCKAP1, RPN1, SLC3A2, and SLC7A11 are among these genes (Liu et al., 2023).

### Immune infiltration analysis

To determine the relative abundances of 22 immune cells in each sample, the CIBERSORT method was used (Supplementary Table S1) (Zhao et al., 2023). The sum of the 22 immune cells proportions in each sample was 1, and *p* < 0.05 represented a significant correlation. We assessed the correlations between the proportional percentage of immune cells and the expression of the DRGs using the spearman correlation coefficient. Histograms, heat maps, and box plots were plotted using the “ggplot2” R packages (version 0.92).

### Unsupervised clustering of NAFLD patients

Based on DE-DRGs, unsupervised clustering analysis of NAFLD patients was performed by using the R package “ConsensusClusterPlus” (version 2.60) (Wilkerson and Hayes, 2010). Cumulative distribution function (CDF) curves, consensus matrix, and consistent cluster score were used to estimate the optimal cluster number.

### Gene set variation analysis

The “c2. cp.Kegg.symbols” and “c5. go.symbols” files from the MSigDB database were first downloaded for this investigation. The enrichment differences between GO and KEGG pathways were then clarified using a non-parametric unsupervised gene set variation analysis (GSVA) approach by the R package of “GSVA” (version 2.11) (Hänzelmann et al., 2013). *p*-value lower than 0.05 was the cutoff value for the statistical significance term.

### Gene set enrichment analysis

The “clusterProfiler” package (version 3.16.1) was used to perform a Gene Set Enrichment Analysis (GSEA), which was then used to determine whether KEGG pathways had an abundance of relevant genes (Kumar and Futschik, 2007).

### Weighted gene co-expression network analysis

Utilizing the “WGCNA” R package (version 1,70.3), WGCNA analysis was carried out on the training set to look into the connection between gene networks and diseases (Langfelder and Horvath, 2008). To ensure the accuracy of the study, we first grouped the samples and eliminated outliers. A soft threshold from 1 to 20 was used for topology calculation to determine the optimal soft threshold. When the minimum module size was set to 100, a “dynamic tree cutting” algorithm was used to group genes with similar patterns into modules. Finally, Pearson correlation analysis was performed to calculate the correlation between modules and traits. Based on the correlation between modules and clinical features, the most relevant module to the disease is selected as the key module.

### Construction of a predictive model based on several machine learning algorithms

Through the use of the “caret” R package (version 6.0.91), we constructed four machine learning models by using data from two distinct DRGs clusters. These models were the random forest model (RF), the support vector machine model (SVM), the generalized linear model (GLM), and the eXtreme Gradient Boosting (XGB) (Nelder and Wedderburn, 1972; Gold and Sollich, 2003; Chen and Guestrin, 2016; Rigatti, 2017). The distinct clusters were considered as the response variable, and the cluster-specific DEGs were selected as explanatory variables. 104 NAFLD samples were randomly classified into training and testing cohort. All these machine learning models were using default parameters and evaluated by 5-fold cross-validation. The caret package (Long and Yang, 2020) was used to automatically adjust the parameters of these models. The aforementioned four machine learning models are analyzed by using the “DALEX” R package (version 2.4.0) to illustrate the relevance of features across these machine learning models as well as the distribution of residuals. The “pROC” R package (version 1.18.0) was used to visualize the area under the ROC curve. As a result, we determined the top machine-learning models for NAFLD and selected significant prognostic genes linked to NAFLD for additional research. The diagnostic utility of this diagnostic model was subsequently validated using ROC curve analysis on the GSE63067 and GSE164760 datasets.

### Construction and validation of the nomogram model

Using the R package “rms” (version 6.2.0) and the screened model genes, we built a diagnostic nomogram model and then used calibration curves and DCA to evaluate the nomogram model’s predictive ability.

### Animal models

Twenty-four 6-week-old male C57BL/6J mice were kept in conventional housing (room temperature: 23°C ± 2°C; 12-h light/dark cycle) with unrestricted access to food and water. Twelve mice each were randomly assigned to the normal chow (NC) and high-fat diet (HFD) groups after 1 week of acclimatization, and each group received either the high-fat diet (60 kcal% fat; d12492, medicine, Jiangsu, China) or standard laboratory food. After 12 weeks of HFD feeding, a mouse model with non-alcoholic fatty liver was created (Dusabimana et al., 2021; Qian et al., 2021). All mice were anesthetized using 2% isoflurane after 12 weeks. After mice were sacrificed by cervical dislocation, blood samples and liver tissues were collected. All studies were approved by the Professional Committee for Animal Protection of Harbin Medical University (2022-DWSYLLCZ-20).

### Histology, and liver triglyceride levels

For histological analysis, formalin-fixed mouse liver tissues were processed and 5-μm-thick paraffin sections were cut and stained with hematoxylin-eosin (H&E). Triglyceride (TG) content in the liver was measured by a commercial kit (A110-1-1; Jiancheng, Nanjing, China) according to the manufacturer’s instructions.

### Quantitative RT-PCR analysis

TRIzol reagent (Invitrogen, Carlsbad, CA, USA) was used to extract total RNA from homogenized tissues. Then, 2*SYBR Green qPCR (Vazyme, Nanjing, China) was used to analyze gene expression after 1 ug of total RNA was reverse transcribed using PrimeScript reverse transcriptase (Takara, Kusatsu, Japan). The expression of β-actin was taken as the control. In Supplementary Table S2, the primer sequences are presented.

### Data analysis

R software (version 4.2.0) was used to conduct bioinformatics analyses. The software GraphPad Prism version 9.0 was used to statistically analyze and visualize the data from animal experiments. Using an unpaired Student’s t-test, the means of two groups of normally distributed variables were compared. Data are presented as the mean ± standard deviation. *p* < 0.05 were considered significantly different.

## Results

### Dysregulation of disulfidptosis regulators and activation of the immune responses in NAFLD patients

**FIGURE 2:**
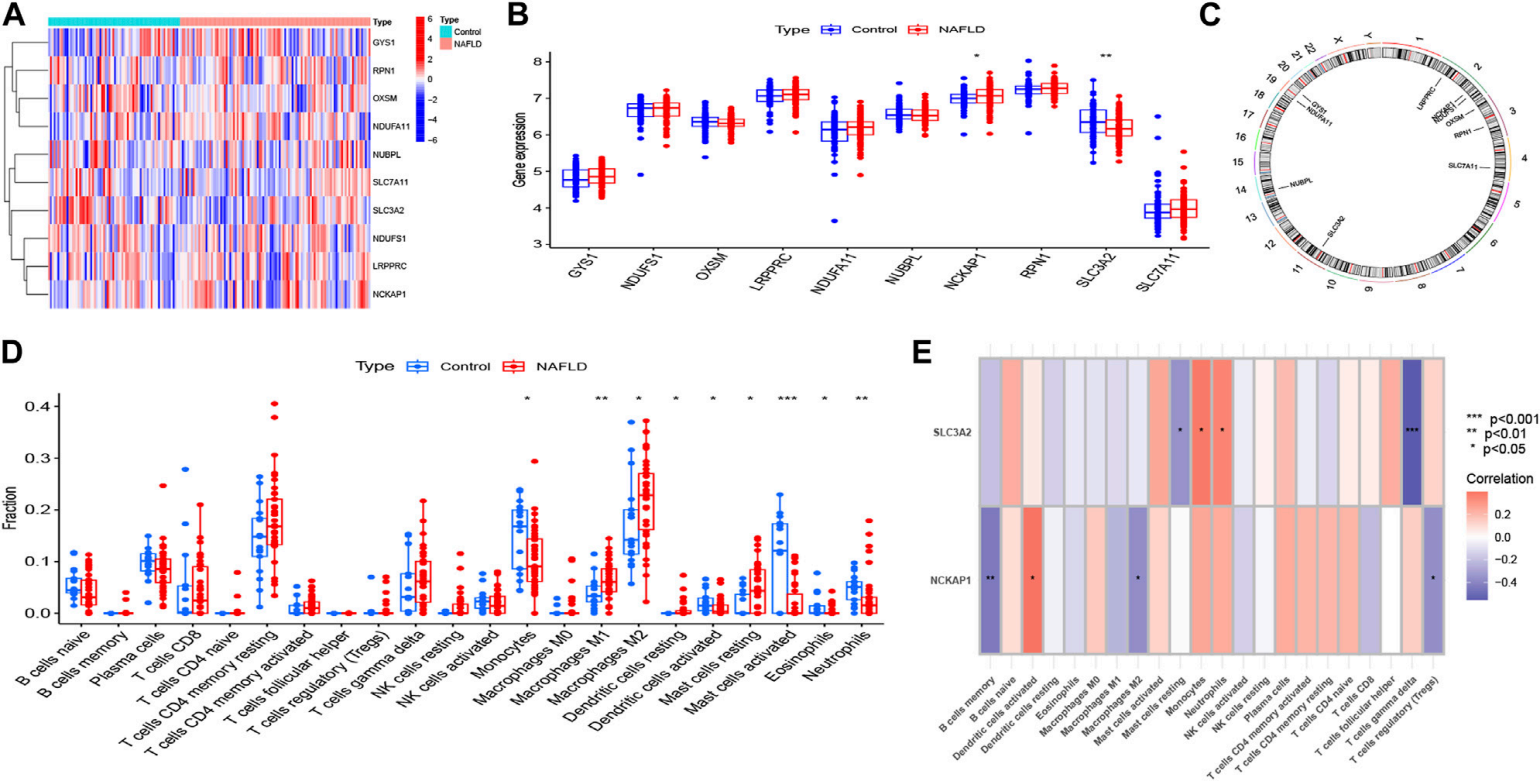
Expression profile of disulfidptosis-related genes (DRGs) in NAFLD. **(A)** Heatmap showing the expression patterns of 10 DRGs in NAFLD and normal samples. **(B)** Boxplots showing the expression of DRGs between NAFLD and control groups. **(C)** The location of 10 DRGs on chromosomes. **(D)** Histogram showing the distribution of 22 immune cells infiltration between the NAFLD group and control groups. **(E)** Correlation analysis of 2 differentially expressed DRGs with 22 immune cells. *p < 0.05, **p < 0.01, ***p < 0.001.

First, we merged and normalized three GEO datasets: GSE89632, GSE48452, and GSE66676 (Supplementary Figures S1, S2). Subsequently, we investigated the expression patterns of the 10 DRGs in control and NAFLD samples (Figure 2A), and the results showed that NCKAP1 was significantly highly expressed in NAFLD samples, while SLC3A2 was lowly expressed in NAFLD samples (Figure 2B). Also, the chromosomal positions of the 10 DRGs were visualized (Figure 2C).

Previous research indicates a direct connection between NAFLD and the immunological microenvironment (Huby and Gautier, 2022). To investigate the variations in the immune microenvironment between NAFLD patients and normal samples, we applied the CIBERSORT algorithm. As seen in Figure 2D, compared to normal samples, NAFLD samples had reduced proportions of monocytes, activated dendritic cells, activated mast cells, eosinophils, and neutrophils, as well as higher proportions of M1 macrophages, M2 macrophages, resting dendritic cells, and resting mast cells. This raises the possibility that immune system changes may be the primary cause of NAFLD. Additionally, Figure 2E’s correlation analysis revealed that NCKAP1 was considerably negatively connected with memory B cells, M2 macrophages, and regulatory T cells, and significantly positively correlated with activated dendritic cells. While SLC3A2 was strongly inversely connected with resting mast cells and δγT cells, it was considered positively correlated with neutrophils and monocytes. This data suggests that the altered immune milieu of NAFLD may be significantly influenced by NCKAP1 and SLC3A2, which act as disulfidptosis molecules in NAFLD patients.

### Identification of disulfidptosis clusters in NAFLD

**FIGURE 3:**
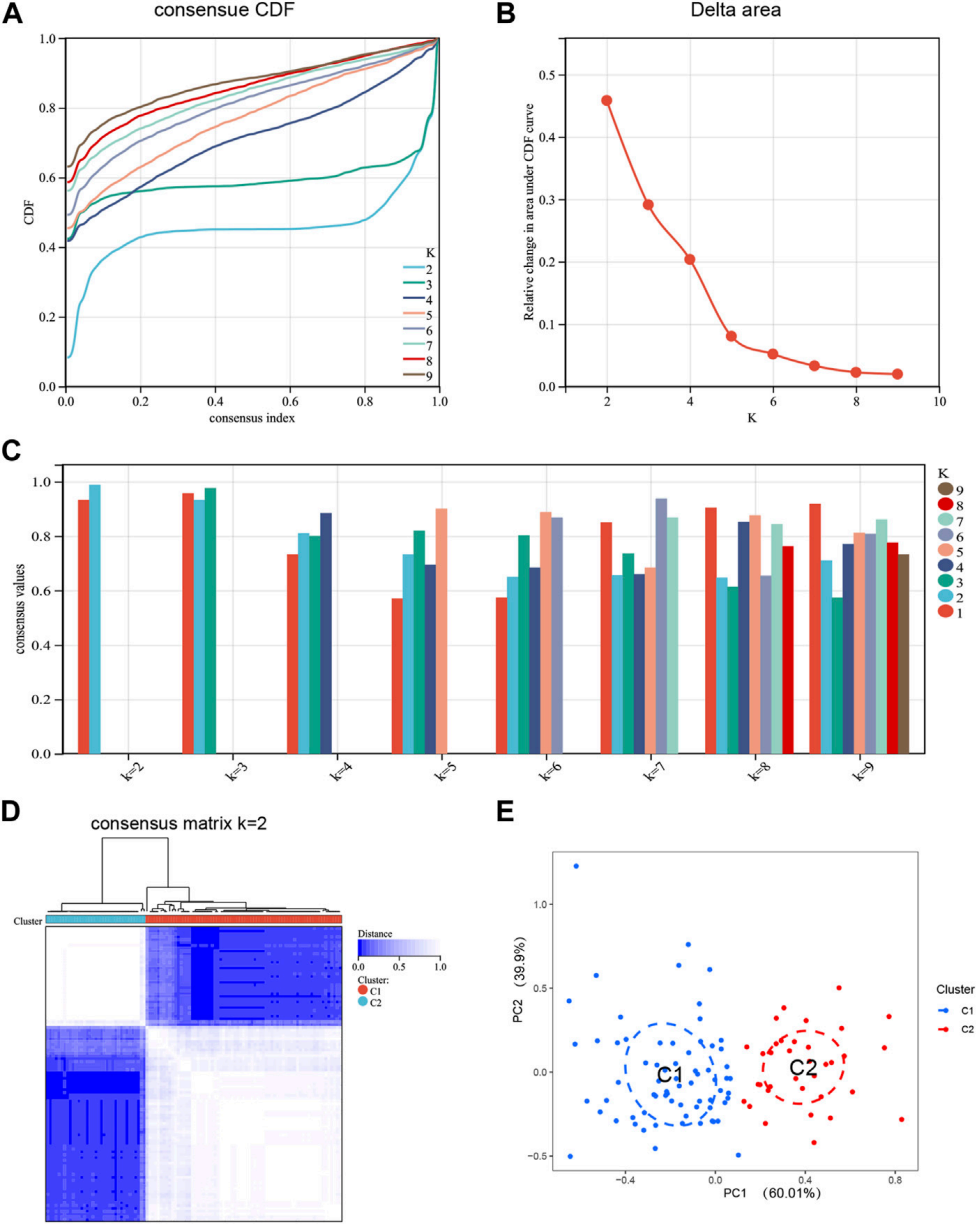
Identification of disulfidptosis-related molecular clusters in NAFLD. **(A)** Cumulative Distribution Function (CDF) plot of consensus clustering, showing the curve of the CDF as the number of clusters changes. **(B)** Delta Area plot, calculating the relative change in the area under the curve (AUC) of the CDF as the number of clusters increases. **(C)** Cluster-consensus plot showing the cluster-consensus value with different k values. The higher the value, the more stable the subtype. **(D)** Consensus matrix plots depicting consensus values on a white to blue color scale ordered by consensus clustering when k = 2. **(E)** PCA plot showing that DE-DRGs effectively classify NAFLD patients into two subgroups. Blue represents Cluster 1; red represents Custer 2.

To elucidate the expression patterns associated with DRGs in NAFLD, we grouped the NAFLD samples based on DE-DRGs using a consensus clustering algorithm. When k = 2, examination of the Delta area and the CDF value showed that the results of clustering was relatively stable (Figure 3A, B). The highest consensus values (all over 0.9) were observed for each subtype when k = 2 (Figure 3C). The consensus matrix showed that each sample in the cluster exhibits strong correlation when k = 2, when the samples in the two subtypes are most stable (Figure 3D). Therefore, k = 2 was the best choice. To verify the differences between Cluster 1 and Cluster 2 samples, PCA analysis showed significant differences between these subtypes (Figure 3E). Finally, based on the expression of DE-DRGs, we categorized the 104 NAFLD samples into two different subtypes, including Cluster 1 (n = 69) and Cluster 2 (n = 35).

### Identification of the immunological microenvironment and biological function in different DRGs clusters

**FIGURE 4:**
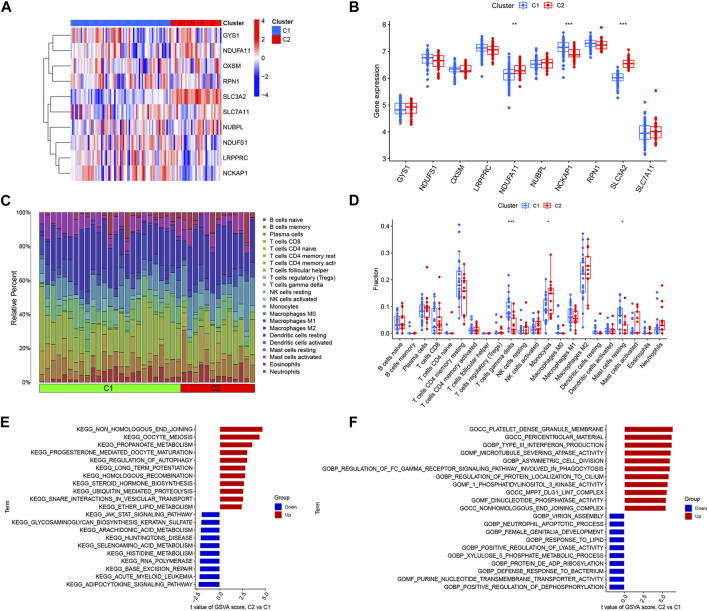
Identification of immune infiltration and biological functional features in DRGs clusters. **(A)** Heat map showing the expression profiles of 10 DRGs in two clusters. **(B)** Box plot showing the expression differences of 10 DRGs between two DRGs clusters. **(C)** Relative abundance of 22 immune cells between two DRGs clusters. **(D)** Box plot showing immune infiltration differences between DRGs clusters. **(E)** Differences in hallmark pathway activities between Cluster1 and Cluster2 samples ranked by t-value of GSVA method. **(F)** Differences in biological functions between Cluster1 and Cluster2 samples ranked by t-value of GSVA method. *p < 0.05; **p < 0.01, ***p < 0.001.

To investigate the molecular differences between clusters, we completely analyzed the DRGs expression differences between Cluster 1 and Cluster 2. We found that the two clusters had distinct DRGs expression landscapes (Figure 4A). Meanwhile, we analyzed the differences in DRGs between different DRGs clusters and found that SLC3A2 and NDUFA11 were upregulated in Cluster 2 and NCKAP1 was downregulated in Cluster 2 (Figure 4B). We then examined the variations in immune cells and their immune activities across various clusters to further study the differences in immune microenvironment features between various DRGs clusters. The findings demonstrated that the immunological microenvironment between Cluster 1 and Cluster 2 was different. (Figure 4C). Cluster 1 had relatively high levels of resting mast cells and γδ T cells; whereas monocytes had higher levels of abundance in Cluster 2 (Figure 4D).

We also performed a GSVA analysis to explore the functional differences between the two clusters. Functional enrichment results showed that non-homologous end-joining, oocyte meiosis, propanoate metabolism, and regulation of autophagy pathways were enhanced in Cluster 2, while the Adipocytokine signaling pathway, JAK-STAT signaling pathway, and amino acid metabolic pathway were upregulated in Cluster 1 (Figure 4E). The results showed that Cluster 2 was closely associated with type III interferon production, asymmetric cell division, and regulation of protein localization to the cilium. However, the neutrophil apoptotic process, positive regulation of dephosphorylation, defense response to the bacterium, and response to lipids were enriched in Cluster 1 (Figure 4F). Therefore, we hypothesize that Cluster 1 may be involved in various immunoregulatory and energy metabolic responses.

### Building co-expression networks and screening gene modules

**FIGURE 5:**
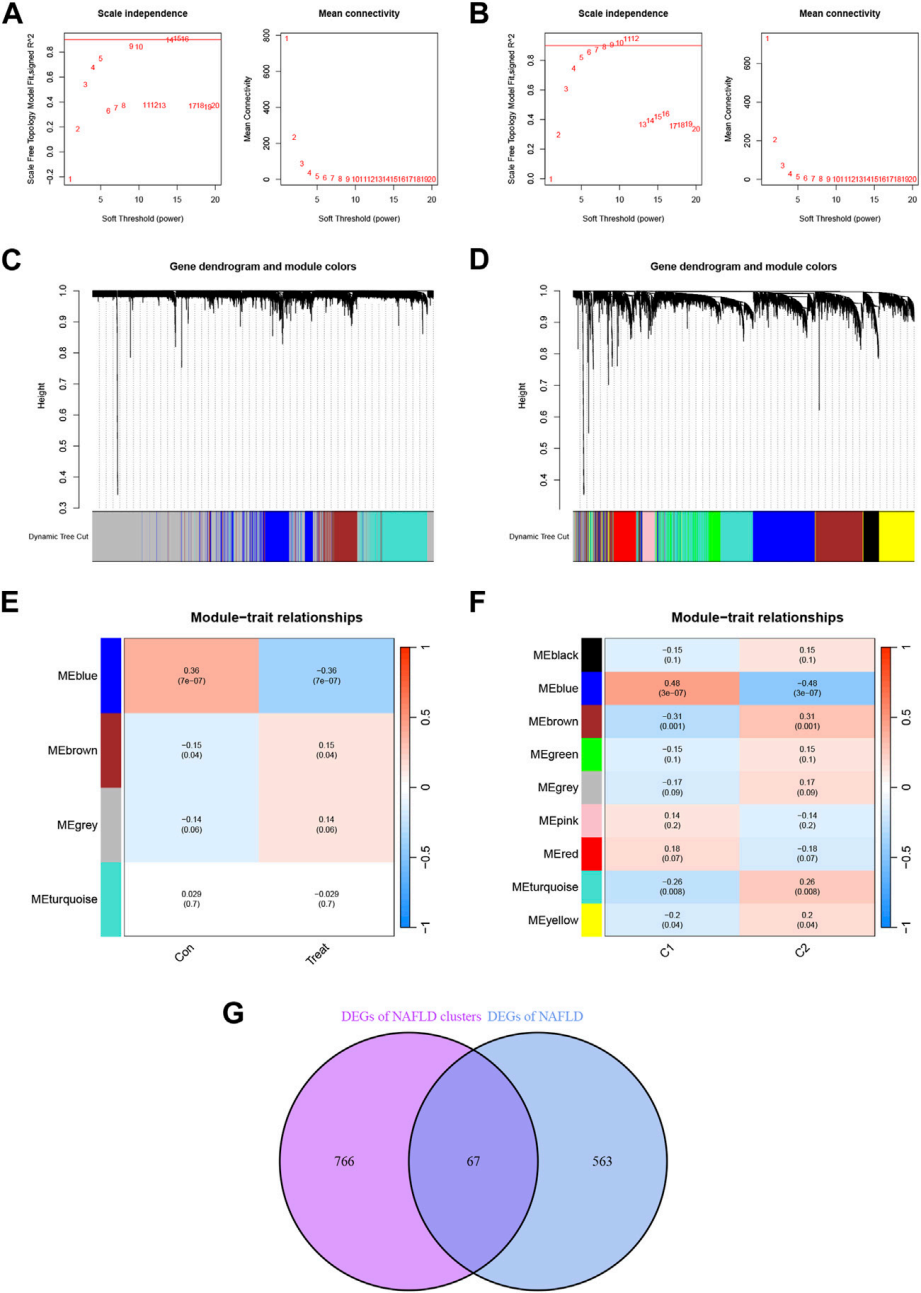
Identification of NAFLD disulfidptosis cluster-specific DEGs. **(A)** Determination of Soft Threshold power for the training set. **(B)** Determination of Soft Threshold power for the disulfidptosis cluster. When scale-free distribution is reached, the optimal soft-power value for training set is 14 while for the disulfidptosis clusters it was 6. **(C)** The origin and merge modules shown under the clustering tree for the training set. **(D)** Origin and merge modules shown under the clustering tree for the disulfidptosis clusters. The clustering dendrogram shows the clustering process of the gene modules. **(E)** Heatmap of the correlation between module eigengenes and the occurrence of NAFLD. **(F)** Heatmap of the correlation between module eigengenes and the disulfidptosis clusters. The values in the small cells of the graph represent the two-calculated correlation values cor coefficients between the eigenvalues of each trait and each module as well as the corresponding statistically significant *p*-values. **(G)** Crossover between module-associated genes in the disulfidptosis cluster and module-associated genes in the training set.

In addition, we analyzed key gene modules closely associated with disulfidptosis clusters using the WGCNA algorithm. All samples were clustered in the dataset and no samples were excluded (Supplementary Figures S3, S4). The value of the optimal soft power for the training set according to the WGCNA method was 14 (Figure 5A), while for the samples related to the disulfidptosis clusters was 6 (Figure 5B). And based on the co-expression network with the optimal soft power, 4 modules were identified in the training set, including MEblue, MEbrown, MEgrey, and MEturquise (Figure 5E) and visualized by hierarchical clustering (Figure 5C). In the disulfidptosis clusters, 9 modules including MEblack, MEblue, MEbrown, MEgreen, MEgrey, MEpink, MEred, MEturquise and MEyellow were identified (Figure 5F) and visualized by hierarchical clustering (Figure 5D). The blue module showed the strongest association with NAFLD. The blue module had the strongest correlation with the disulfidptosis clusters. By analyzing the interaction between module-associated genes of disulfidptosis clusters and module-associated genes of NAFLD and non-NAFLD people, a total of 67 cluster-specific DEGs were discovered (Figure 5G; Supplementary Table S3).

### Construction and evaluation of machine learning models

**FIGURE 6:**
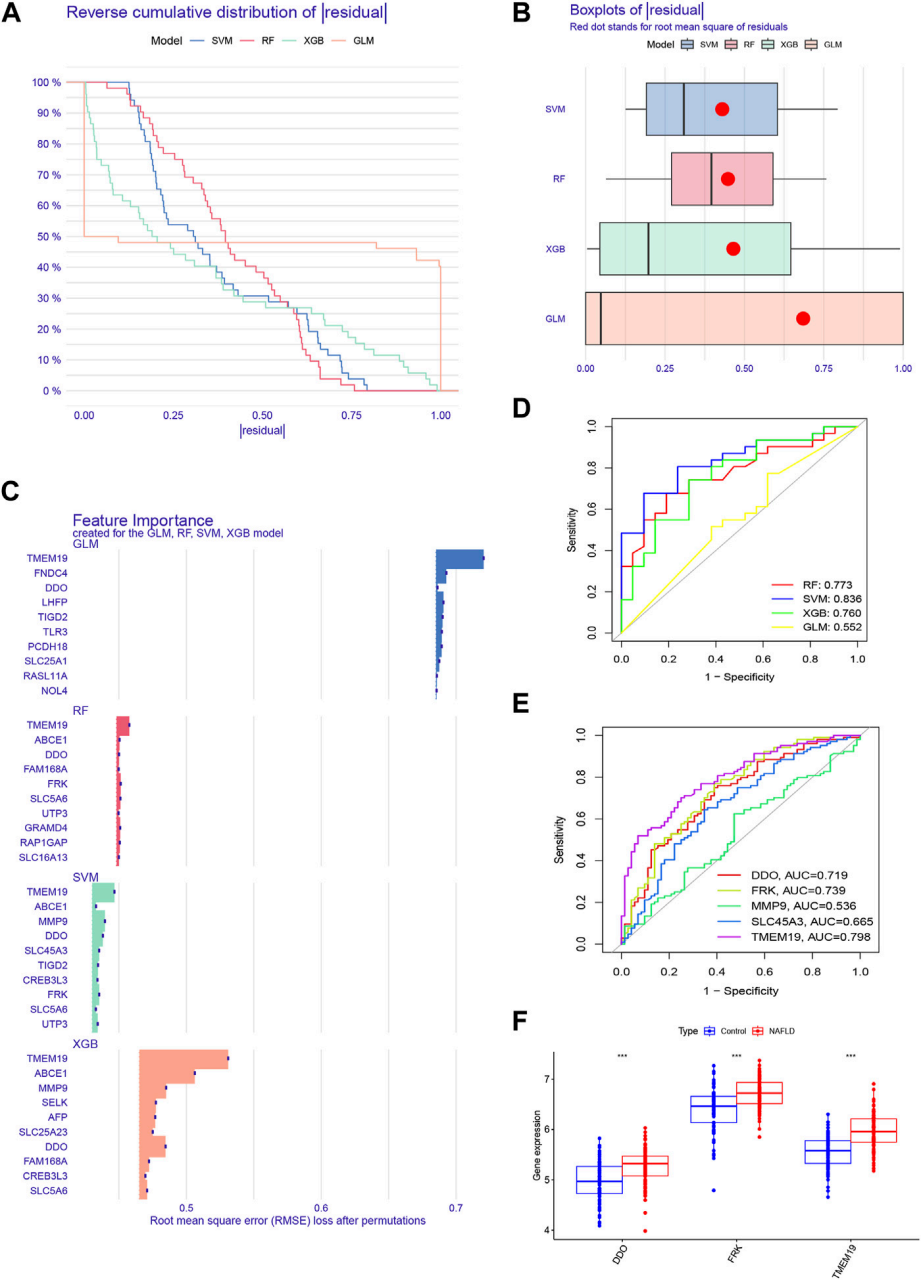
Construction and evaluation of four machine models. **(A)** Cumulative residual distribution of each machine learning model. **(B)** Boxplots showed the residuals of four machine learning models. Red dot represented the root mean square of residuals (RMSE). **(C)** The important features in four machine models. **(D)** ROC analysis of each machine learning model based on 5-fold cross-validation in the testing cohort. **(E)** ROC analysis of DDO, FRK, MMP9, SLC45A3, and TMEM19 in the training set. **(F)** Boxplots showed the expression of DDO, FRK and TMEM19 between NAFLD and control groups in the training set.

Based on the expression profiles of 67 cluster-specific DEGs, we constructed four machine-learning models to further uncover subtype-specific genes with high diagnostic values. The outcomes demonstrated that the residuals for the SVM and RF machine learning models were reasonably low (Figures 6A, B). By computing the receiver operating characteristic (ROC) curves, we also assessed the diagnostic performance of the four machine learning algorithms in the testing cohort. The root means square error (RMSE) was then used to order the top 10 significant feature variables in each model (Figure 6C). The SVM machine learning model, which had an AUC of 0.836 compared to RF’s 0.773, XGB’s 0.760, and GLM’s 0.552, had the highest AUC (Figure 6D). Combining these findings, the SVM model was found to be the most effective at distinguishing patients with various clusters among the four machine-learning models. We chose the top five predicted genes (MMP9, DDO, SLC45A3, FRK, and TMEM19) from the SVM model for additional investigation. DDO, FRK, and TMEM19 were three of the five genes with AUC values larger than 0.7 (Figure 6E), and these three genes were considerably elevated in NAFLD in comparison to controls (Figure 6F). We also compared their expression differences between disulfidptosis-associated clusters and the result showed that the expression of all three genes was greater in Cluster 1 than in Cluster 2, but the expression of FRK and TMEM19 was significantly different between Clusters 1 and 2, while the expression of DDO was not significantly different between disulfidptosis-associated clusters (Supplementary Figure S5).

### Establishment of a nomogram for predicting NAFLD

**FIGURE 7:**
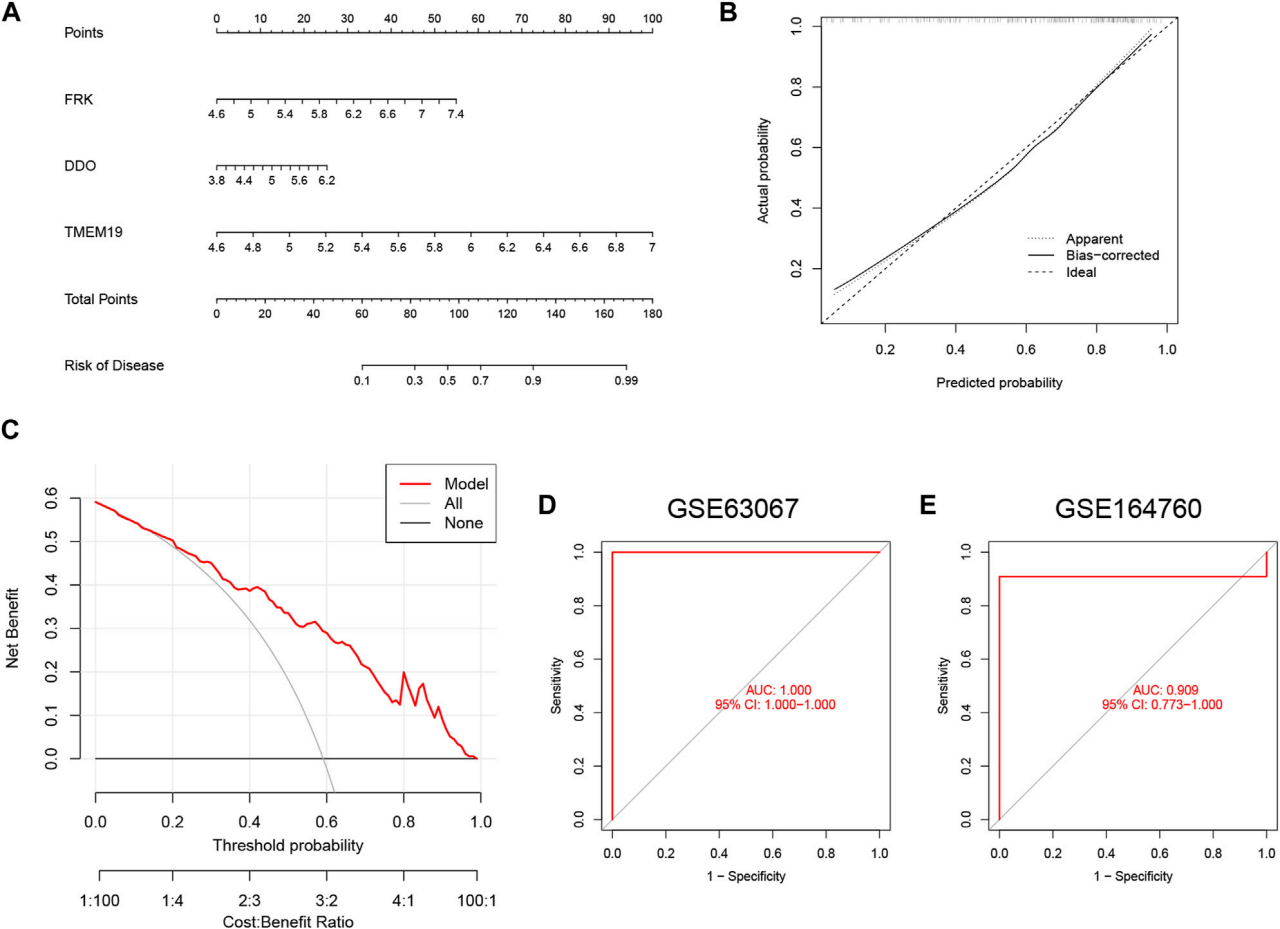
Construction of the nomogram and validation of the 3-gene-based SVM model. **(A)** Construction of a nomogram to predict the risk of NAFLD based on the 3-gene-based SVM model. **(B)** Calibration curves for estimating the prediction accuracy of the nomogram. **(C)** DCA showed the clinical benefit of nomogram. **(D,E)** ROC analysis of 3-gene-based SVM model in GSE63067 **(D)** and GSE164760 **(E)** datasets.

To better predict the risk of patient morbidity, we constructed a nomogram model based on the three genes in the SVM model (Figure 7A). The results of calibration curves showed that the predictive ability of the nomogram model was accurate (Figure 7B). In addition, patients with NAFLD could benefit from column line graphs as shown in the DCA (Figure 7C). After that, we tested our 3-gene prediction model on two independent liver tissue datasets to validate its accuracy. The ROC curves demonstrated that the performance of the 3-gene prediction model was satisfactory, with an AUC value of 1.000 in the GSE63067 dataset (Figure 7D), and 0.909 in the GSE164760 dataset (Figure 7E). This indicates that our diagnostic model is equally effective in differentiating NAFLD patients from normal individuals.

### Functional enrichment and correlation analysis between biomarkers and immune cells

**FIGURE 8:**
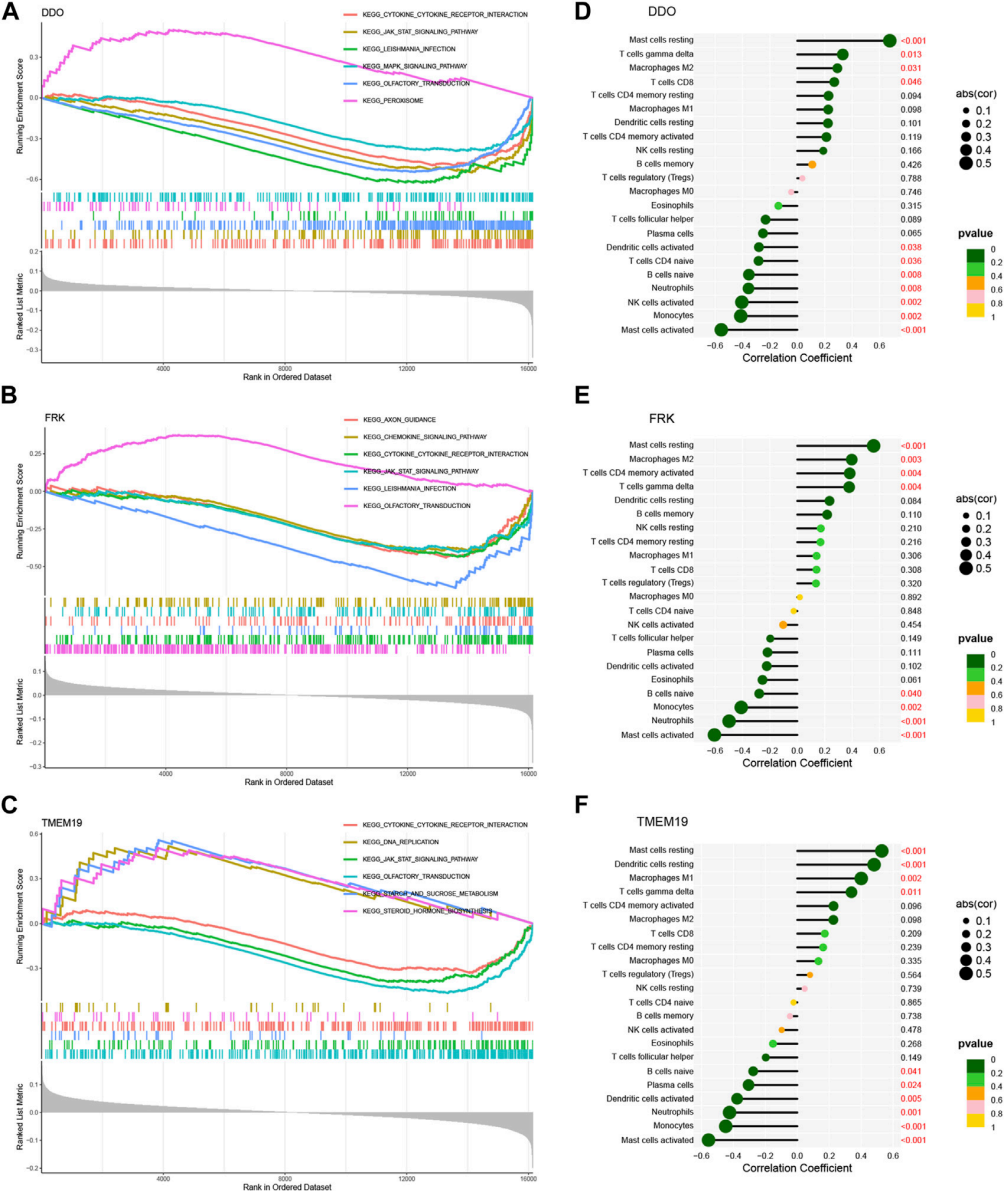
GSEA of 3 model genes and correlation analysis with immune cells. GSEA of DDO **(A)**, FRK **(B)** and TMEM19 **(C)** genes using KEGG gene sets. Correlation analysis of DDO **(D)**, FRK **(E)**, TMEM19 **(F)** gene expression and 22 infiltrating immune cells. The strength of the link between genes and immune cells is shown by the size of the dots. The bigger the dot, the stronger the link. The *p*-value is shown by the color of the dots. The more green the color, the lower the *p*-value. *p*-value <0.05 is considered to be statistically significant.

To further investigate the potential role of the three biomarkers in NAFLD, we performed GSEA on each biomarker in the training set. The results of GSEA showed that the “cytokine receptor interaction” pathway and the “JAK-STAT signaling pathway” were enriched in the groups with low expression of DDO, FRK, and TMEM19 (Figures 8A–C).

Next, we analyzed whether or not there was a connection between the expression of three diagnostic genes and infiltrated immune cells in the training set. The results showed that the DDO gene was positively correlated with resting mast cells, δγT cells, M2 macrophages, and CD8T cells; and negatively correlated with activated mast cells, monocytes, activated NK cells, neutrophils, naive B cells, naive CD4 T cells and activated dendritic cells (Figure 8D). And FRK gene was positively correlated with resting mast cells, M1 macrophages, resting dendritic cells, and δγT cells; and negatively correlated with activated mast cells, neutrophils, monocytes, and naive B cells (Figure 8E). TMEM19 gene was positively correlated with naive CD4 T cells, resting dendritic cells, M1 macrophages, andδγT cells; and it was negatively correlated with activated mast cells, neutrophils, monocytes, naive B cells, activated dendritic cells, and Plasma cells (Figure 8F). In general, the expression of these genes may be related to the amount of infiltration of various immune cells, which suggests that these critical diagnostic genes may be engaged in immune control in the pathogenesis of NAFLD.

### Validation of model genes in mouse models

**FIGURE 9:**
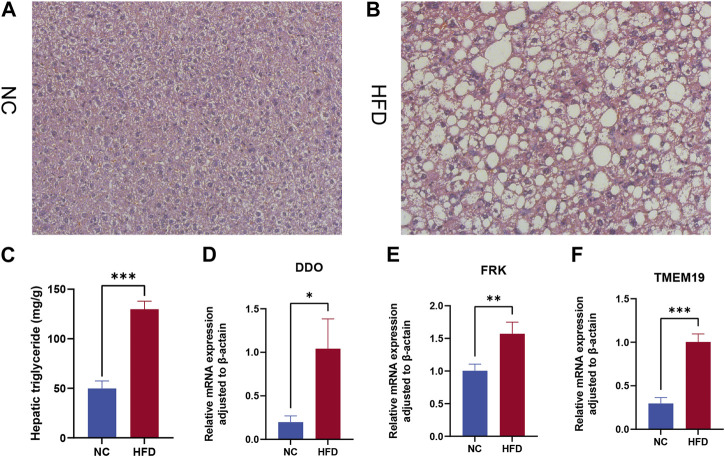
Expression levels of model gene mRNA were verified in the HFD mouse model. β-Actin was controlled. **(A,B)** H&E staining of livers from control **(A)** and high-fat diet (HFD) fed mice **(B)**. Magnification ×400. **(C)** Hepatic triglyceride (TG) concentrations. **(D–F)** Relative mRNA levels of **(D)** DDO, **(E)** FRK, and **(F)** TMEM19. Relative mRNA levels were normalized to those of β-actin. Values are shown as the mean ± s.d. *p < 0.05; **p < 0.01, ***p < 0.001.

We assessed the expression levels of these biomarkers in a 12-week HFD-fed mice model to further confirm the validity of the biomarkers discovered in NAFLD. The HFD group had severe hepatic steatosis with sporadic inflammation, as seen by H&E staining of liver tissue sections (Figures 9A, B). In comparison to the NC group, the levels of hepatic TG were considerably higher in the HFD group (Figure 9C). Finally, we looked at three model genes’ expression in the livers of HFD and NC groups. We discovered that the HFD group had significantly higher levels of DDO, FRK, and TMEM19 expression than the NC group (Figures 9D–F).

## Discussion

NAFLD is the most prevalent liver and metabolic disease worldwide, which has a serious impact on public health (Chalasani et al., 2018). Lifestyle measures to lose weight are still the most effective therapy for NAFLD since the condition’s existing therapies are insufficiently effective (2016). After changing one’s lifestyle, medication may still be needed to address NAFLD. Fortunately, progress has been made in the study of insulin sensitizers for the treatment of fatty liver disease [30]. More critically, for the diagnosis and treatment of NAFLD, novel therapeutic targets are consistently uncovered (Pan et al., 2021; Qu et al., 2021). Therefore, finding better molecular clusters is crucial to determining how to treat NAFLD on an individual basis. Liu’s concept of “disulfidptosis” sheds fresh light on how dysregulated glucose metabolism and disulfide play a part in cell death (Liu et al., 2023). However, further research has not been done on the precise processes of disulfidptosis and its regulation function in many diseases. We aimed to clarify the precise functions of genes related to disulfidptosis in the NAFLD phenotype and immunological microenvironment. Furthermore, NAFLD subtypes were predicted using genetic markers linked to disulfidptosis.

In this study, we comprehensively analyzed the expression of DRGs in the liver of NAFLD patients versus normal controls. The expression of DRGs in NAFLD patients was significantly different compared to normal subjects, suggesting an important role of DRGs in the development of NAFLD. Subsequently, our immune infiltration revealed an altered abundance of immune cells between controls and patients with NAFLD. Higher levels of M1 macrophage, M2 macrophage, resting dendritic cell, and resting mast cell infiltration were seen in patients with NAFLD, while lower levels of monocyte, activated dendritic cell, activated mast cell, eosinophil, and neutrophil infiltration were seen in these patients. These findings were consistent with previous studies that examined liver tissues (Wen et al., 2021; Zhang et al., 2022). In addition, we used unsupervised clustering analysis to illustrate the expression landscape of different disulfidptosis regulatory patterns underlying NAFLD patients based on DE-DRGs. Examination of the consensus matrix and the CDF value showed that the number of subtypes was relatively stable when k = 2. Also based on the consensus values, two distinct disulfidptosis-related clusters were identified, which will provide new insights for individualized treatment of NAFLD. We also found that two DRGs (SLC3A2 and NDUFA11) were upregulated in Cluster 2 and NCKAP1 was downregulated in Cluster 2. Cluster 1 exhibited relatively high levels of immune infiltration. Cluster-specific DEGs indicated that the Adipocytokine signaling pathway, JAK-STAT signaling pathway, and amino acid metabolic pathway were upregulated in Cluster 1. Adipokines are peptides widely found in adipose tissue and usually play an important role in the pathogenesis of NAFLD by regulating hepatic fat accumulation, insulin resistance, and inflammatory responses in an autocrine, paracrine, and endocrine manner (Polyzos et al., 2016). The adipocytokine signaling pathway has also been reported to be important for immune cell activation and differentiation (Wilk et al., 2011). The JAK-STAT signaling pathway is a classical inflammatory regulatory pathway that plays a key role in the inflammatory response and macrophage activation in particular (Hu et al., 2007). Therefore, we have reason to believe that Cluster 1 may have a higher level of immune infiltration through the Adipocytokine signaling pathway, the JAK-STAT signaling pathway.

Due to machine learning’s improved prediction performance, lower error rates, and increased dependability, it has become more and more common to diagnose NAFLD and screen important genes and immune cells in recent years (Bao et al., 2023; Zhang et al., 2023). As a result, we evaluated the four machine learning models’ effectiveness in making predictions. In the training set, the SVM machine learning technique had the best prediction efficacy (AUC = 0.836), confirming its successful prediction of NAFLD. Among the top five significant genes of the SVM machine learning method, DDO, FRK, and TMEM19 showed significant prediction performance and they were significantly upregulated in NAFLD samples. Therefore, we selected DDO, FRK, and TMEM19 to construct the SVM model. DDO encodes a d-aspartate oxidase that specifically acts on free acidic d-amino acids (Zaar et al., 2002). This d-amino acid may function as an endogenous NMDA receptor agonist, which is important in neurodevelopmental problems and is substantially enriched in the brain before birth (Molinaro et al., 2010; Molla et al., 2020). Recent research indicates a connection between DDO and schizophrenia (Lombardo et al., 2022). Additionally, NAFLD, a multisystem illness, has a significant co-morbidity rate with psychological disorders (Gangopadhyay et al., 2022). DDO may be a significant factor connecting these two diseases. This may assist in understanding the processes behind the connection between NAFLD and mental diseases. FRK is also known as protein tyrosine kinase 5 (Goel and Lukong, 2015). It has been discovered that FRK mostly inhibits tumor cell growth in gliomas and breast cancer (Yim et al., 2009); by contrast, FRK contributes to the development of tumors in cases of pancreatic and hepatocellular carcinoma (Goel and Lukong, 2016). And its expression was upregulated in 50% of hepatocellular carcinoma tissues compared to normal liver tissues (Chen et al., 2013). Meanwhile, a previous study found that overexpression of FRK promoted tumor cell proliferation, invasion, and non-adherent cell growth (Chen et al., 2013). Effective inhibitors against FRK, fortunately, have been reported (Li et al., 2010). This might have a big impact on how the disease is treated. As with NAFLD, our research raises the possibility that strategies to reduce FRK expression may aid in the treatment of the condition, although additional clinical and experimental research is still needed to validate this. Additionally, CRISPR-Cas9-mediated gene editing techniques make it possible to spatially control FRK expression, which is critical for the targeted inhibition of FRK gene expression. Little is known about TMEM19, a member of the transmembrane protein family that encodes a transmembrane protein involved in protein binding (Kanamoto et al., 2009). It is significant to note that the “JAK-STAT signaling pathway” was blocked in the high expression groups of DDO, FRK, and TMEM19, according to functional enrichment data. The activation of the JAK-STAT pathway was found to be closely related to the resistance to cellular oxidative stress and the maintenance of mitochondrial function (Ni and Wang, 2016; Ou et al., 2018). Therefore, we propose a hypothesis that these genes promote NAFLD disease progression by inhibiting the JAK-STAT signaling pathway by promoting endoplasmic reticulum oxidative stress damage and apoptosis. However, the regulatory relationships between these key genes and the mechanisms of action of various signaling pathways with NAFLD still need further experimental validation.

The 3-gene-based SVM model could correctly identify NAFLD, according to the external validation datasets GSE63067 (AUC = 1.000) and GSE164760 (AUC = 0.909), which provided additional insight for the diagnosis of NAFLD. More significantly, we then developed a nomogram model for the diagnosis of NAFLD subtypes using DDO, FRK, and TMEM19. The results showed that the actual results in the calibration plots were highly consistent with the predicted results, indicating that the model could provide a valuable reference for the prediction of NAFLD, and the DCA showed that the model had significant clinical utility. Therefore, this model was found to have significant predictive efficacy, demonstrating its applicability in clinical applications.

Finally, we designed in mouse experiments to verify whether the model genes were differentially expressed in the liver of the NAFLD mouse model. Based on the qRT-PCR results, we found that the three model genes DDO, FRK, and TMEM19 were differentially expressed in the HFD group and the control group, and the expression trends were consistent with the results of bioinformatics analysis.

Of course, this study inevitably has some limitations. First, individual differences in the samples in the dataset used in this study may affect the generalizability of the results of the analysis. In addition, only the mRNA level of the model genes, not the protein level, was verified by RT-qPCR. The use of mouse models rather than human samples may affect the validation of mRNA expression differences of model genes in NAFLD disease. Finally, the evidence provided for the validation of the model is weak and more relevant *in vivo* and *in vitro* experiments are needed to demonstrate the role of these model genes and their potential mechanisms in NAFLD.

In conclusion, our study demonstrates a relationship between DRGs and immune cells and clarifies the immune system’s variability across individuals with various disulfidptosis clusters. Combining WGCNA analysis and machine learning models to screen for disease signature genes, SVM model were identified as the optimal prediction models for NAFLD. Three disease signature genes, DDO, FRK and TMEM19, were predicted. And their mRNA expression levels were validated in the NAFLD model. The prognostic model based on the 3 genes may provide a new approach to predicting the prognosis of NAFLD.

## Data Availability

The original contributions presented in the study are included in the article/Supplementary Material, further inquiries can be directed to the corresponding author.
