# Supplementary material for: Identification and validation of disulfidptosis-associated molecular clusters in non-alcoholic fatty liver disease
Source: Front Genet. 2023 Sep 8;14:1251999. doi: 10.3389/fgene.2023.1251999 (PMC10514914; doi:10.3389/fgene.2023.1251999)
Supplement: Supplementary file 4 [file DataSheet1.docx]

Supplementary Material

Identification and validation of disulfidptosis-associated molecular clusters in non-alcoholic fatty liver disease

**Xiaoxiao Yu, Zihao Guo, Zhihao Fang, Kai Yang, Changxu Liu, Zhichao Dong, Chang Liu***

Department of General Surgery, Fourth Affiliated Hospital of Harbin Medical University, Harbin, China

* Correspondence: Chang Liu: changliu@163.com

# Supplementary Figures


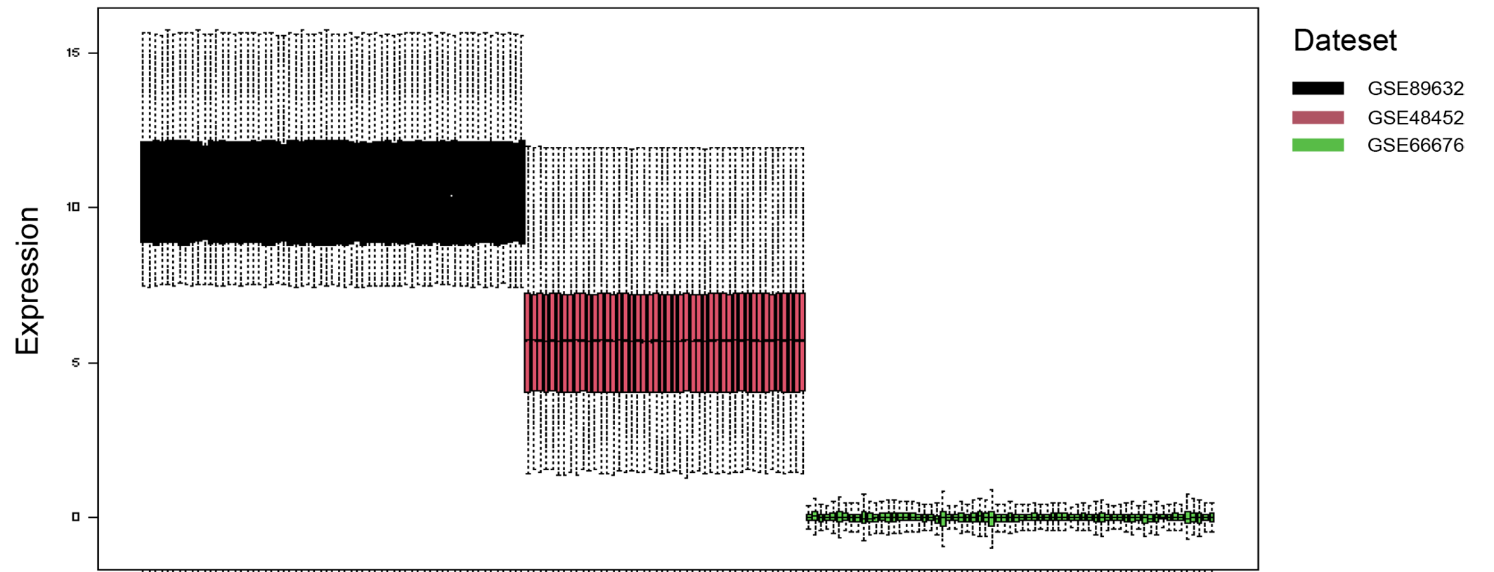


**Supplementary Figure 1. Boxline diagram of the merged dataset before correction.** **Horizontal coordinates represent the list of samples and vertical coordinates represent the gene expression values (log2 transformed). The black group represents dataset GSE89632, the red group represents dataset GSE48452, and the green group represents dataset GSE66676.**

**
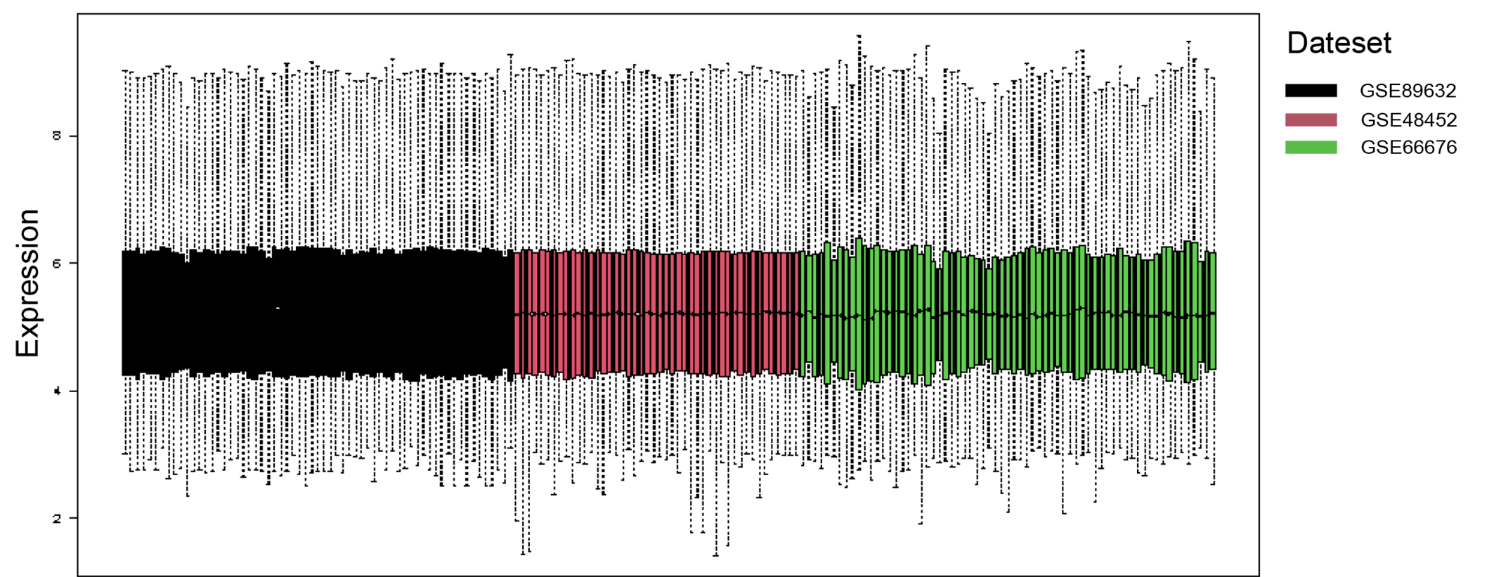
**

**Supplementary Figure 2. Boxline diagram of the combined dataset after correction. Horizontal coordinates represent the list of samples and vertical coordinates represent the gene expression values (log2 transformed).** **The black group represents dataset GSE89632, the red group represents dataset GSE48452, and the green group represents dataset GSE66676.**


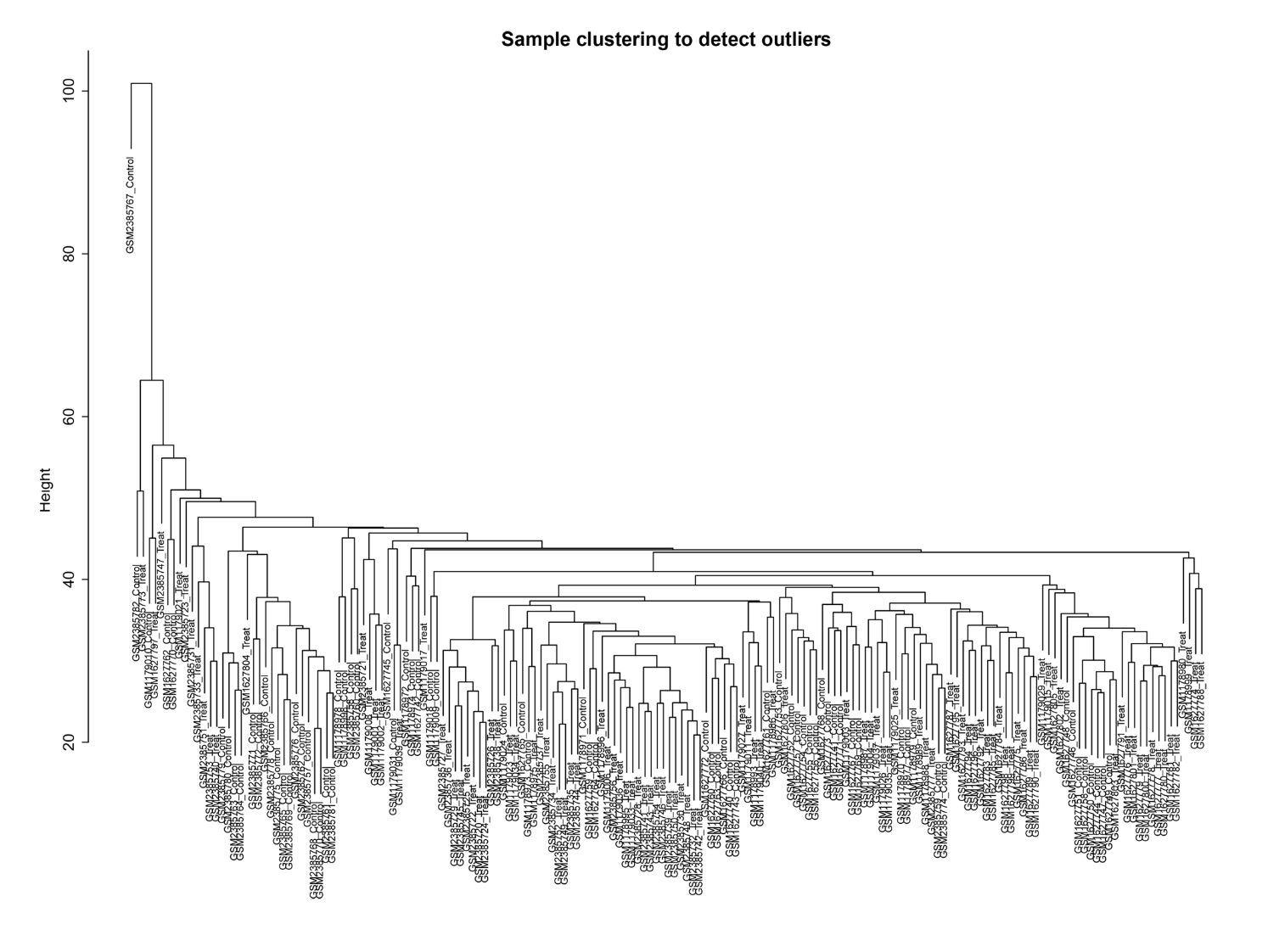


**Supplementary Figure 3**. **Clustering according to the expression levels of control and NAFLD patients in the training set.** **Each branch represents a sample in the data sets, and there is no outlier sample in each data set.**


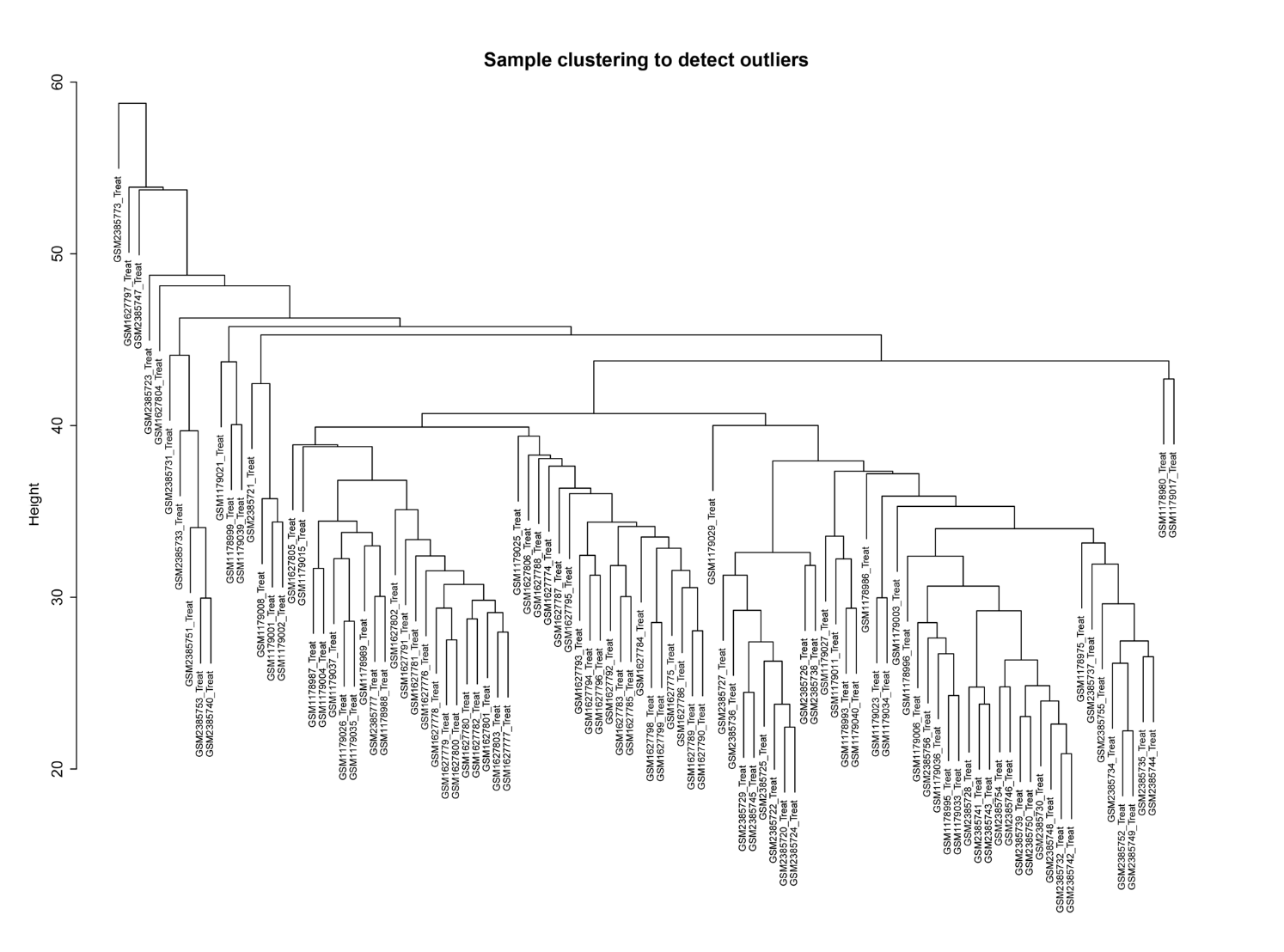


**Supplementary Figure 4. Clustering according to the expression levels of NAFLD patients in the disulfidptosis clusters.** **Each branch represents a sample in the data sets, and there is no outlier sample in each data set.**

#
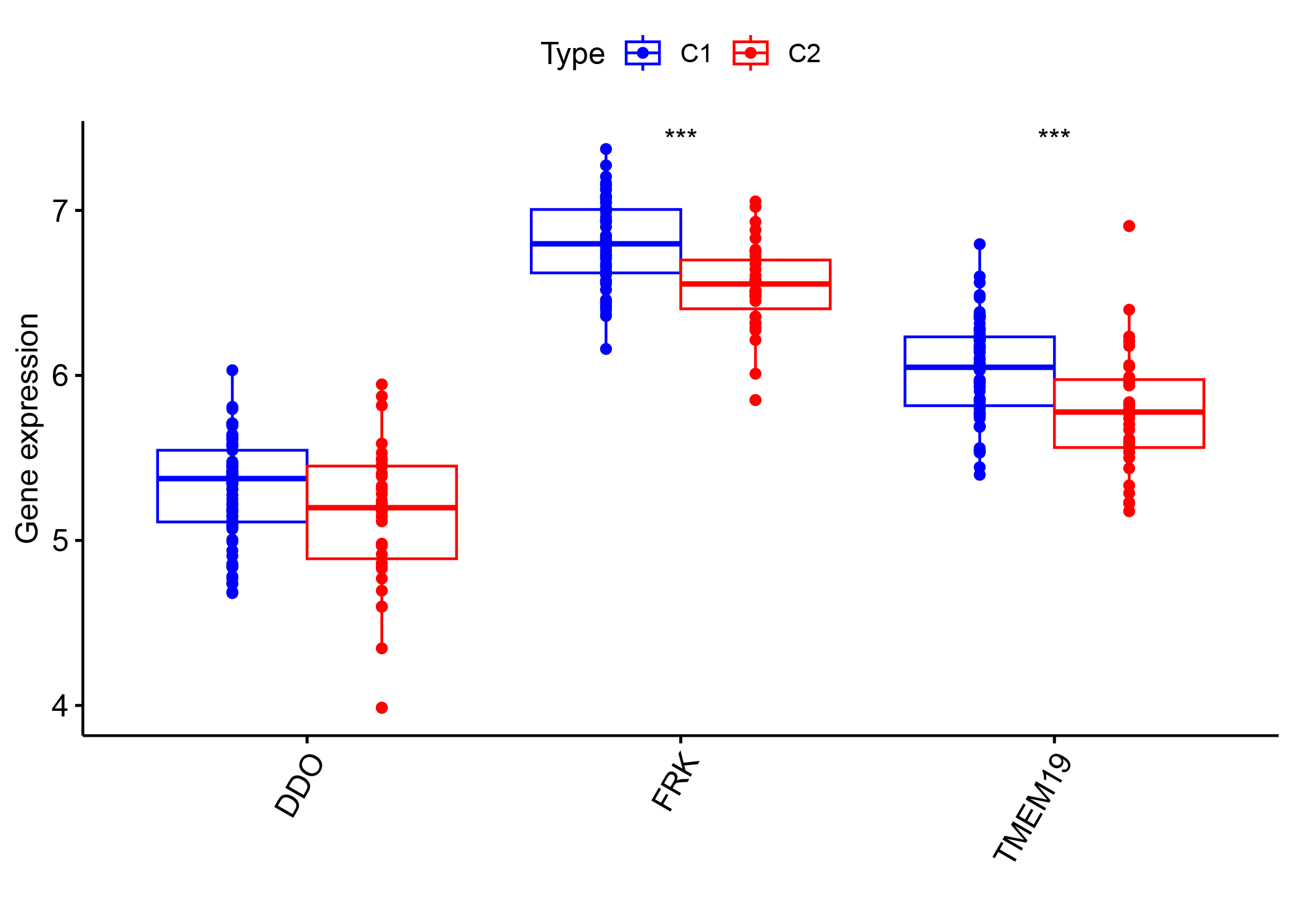


# Supplementary Figure 5. Boxplots showing the expression of DDO、FRK and TMEM19 between Cluster 1 and Cluster 2.
